# Assessment of *In Vitro* and *In Silico* Protocols for Sequence-Based Characterization of the Human Vaginal Microbiome

**DOI:** 10.1128/mSphere.00448-20

**Published:** 2020-11-18

**Authors:** Luisa W. Hugerth, Marcela Pereira, Yinghua Zha, Maike Seifert, Vilde Kaldhusdal, Fredrik Boulund, Maria C. Krog, Zahra Bashir, Marica Hamsten, Emma Fransson, Henriette Svarre-Nielsen, Ina Schuppe-Koistinen, Lars Engstrand

**Affiliations:** a Centre for Translational Microbiome Research, Department of Microbiology, Tumour and Cell Biology, Science for Life Laboratory, Karolinska Institutet, Solna, Sweden; b Department of Medicine Solna, Division of Infectious Diseases, Karolinska University Hospital, Center for Molecular Medicine, Karolinska Institutet, Solna, Sweden; c The Recurrent Pregnancy Loss Unit, Capital Region of Denmark, Rigshospitalet and Hvidovre Hospital, Copenhagen, Denmark; d Department of Clinical Immunology, Copenhagen University Hospital, Copenhagen, Denmark; e Department of Obstetrics and Gynaecology, Holbæk Hospital, Holbæk, Denmark; f Department of Women’s and Children’s Health, Uppsala University, Uppsala, Sweden; g Department of Obstetrics and Gynecology, Hvidovre Hospital, Copenhagen, Denmark; Nanjing Normal University

**Keywords:** 16S rRNA, PCR, amplicon, human microbiome, metagenomics, molecular methods, quantitative methods, vaginal microbiome

## Abstract

The vaginal microbiome has been connected to a wide range of health outcomes. This has led to a thriving research environment but also to the use of conflicting methodologies to study its microbial composition. Here, we systematically assessed best practices for the sequencing-based characterization of the human vaginal microbiome. As far as 16S rRNA gene sequencing is concerned, the V1-V3 region performed best *in silico*, but limitations of current sequencing technologies meant that the V3-V4 region performed equally well. Both approaches presented very good agreement with qPCR quantification of key taxa, provided that an appropriate bioinformatic pipeline was used. Shotgun metagenomic sequencing presents an interesting alternative to 16S rRNA gene amplification and sequencing but requires deeper sequencing and more bioinformatic expertise and infrastructure. We assessed different tools for the removal of host reads and the taxonomic annotation of metagenomic reads, including a new, easy-to-build and -use reference database of vaginal taxa. This curated database performed as well as the best-performing previously published strategies. Despite the many advantages of shotgun sequencing, none of the shotgun approaches assessed here agreed with the qPCR data as well as the 16S rRNA gene sequencing.

**IMPORTANCE** The vaginal microbiome has been connected to various aspects of host health, including susceptibility to sexually transmitted infections as well as gynecological cancers and pregnancy outcomes. This has led to a thriving research environment but also to conflicting available methodologies, including many studies that do not report their molecular biological and bioinformatic methods in sufficient detail to be considered reproducible. This can lead to conflicting messages and delay progress from descriptive to intervention studies. By systematically assessing best practices for the characterization of the human vaginal microbiome, this study will enable past studies to be assessed more critically and assist future studies in the selection of appropriate methods for their specific research questions.

## Abstract (precis)

The vaginal microbiome has been connected to various aspects of host health, including susceptibility to sexually transmitted infections as well as gynecological cancers and pregnancy outcomes. This has led to a thriving research environment but also to conflicting available methodologies, including many studies that do not report their molecular biological and bioinformatic methods in sufficient detail to be considered reproducible. This can lead to conflicting messages and delay progress from descriptive to intervention studies. By systematically assessing best practices for the characterization of the human vaginal microbiome, this study will enable past studies to be assessed more critically and assist future studies in the selection of appropriate methods for their specific research questions.

## INTRODUCTION

The human vaginal microbiome plays a key role in maintaining the gynecological health of women of reproductive age. Estrogen is responsible for the cyclic maturation of the vaginal epithelium and the deposition of glycogen in vaginal epithelial cells (1). Shed glycogen-rich cells are an excellent carbon source for lactic acid bacteria (2). Lactic acid lowers the local pH and has bactericidal and immune regulatory effects (3). In addition to keeping bacterial balance and preventing bacterial vaginosis (BV) and aerobic vaginitis (AV) (4), the vaginal microbiome has been shown to play a protective role against infections with viruses such as human papillomavirus (HPV) (5), herpes simplex virus 2 (HSV-2) (6), and human immunodeficiency virus (HIV) (7). The vaginal microbiome might also be protective against adverse pregnancy outcomes, such as early miscarriage (8) and preterm birth (9), as well as gynecological cancers (10).

In clinical practice, the diagnosis of bacterial vaginosis is often based on experienced vaginal symptoms and pH testing, sometimes combined with a visual assessment of a vaginal smear wet mount under microscopy. Systems such as the Amsel criteria (11) and Nugent scoring (12) have been developed to assist in this assessment but are low resolution and low throughput. In research settings, however, it has become standard to sequence part of the 16S rRNA gene to characterize the vaginal microbiome. However, no consensus exists in this field for experimental or bioinformatic best practices, with different studies (sometimes within the same research group) focusing on different variable regions of the 16S rRNA gene (Table 1) (13–20).

**TABLE 1:** List of primer pairs considered for *in silico* analysis, including region, sequence, and citation

Variable region	Forward primer position	Forward primer sequence	Reverse primer position	Reverse primer sequence	Reference
V1-V2	27f	AGAGTTTGATCCTGGCTCAG	338r	GCTGCCTCCCGTAGGAGT	14, 16
V1-V3	27f	AGAGTTTGATCCTGGCTCAG	534r	ATTACCGCGGCTGCTGG	15
V1-V3	27f-pool	4× AGAGTTTGATYMTGGCTCAG; 1× AGGGTTCGATTCTGGCTCAG; 1× AGAATTTGATCTTGGTTCAG	515r	TTACCGCGGCKGCTGVCAC	13, 17
V1-V3	27f-pool	4× AGAGTTTGATYMTGGCTCAG; 1× AGGGTTCGATTCTGGCTCAG; 1× AGAATTTGATCTTGGTTCAG	534r	ATTACCGCGGCTGCTGG	13
V3-V4	319f	ACTCCTRCGGGAGGCAGCAG	806r	GGACTACHVGGGTWTCTAAT	19
V3-V4	341f	CCTACGGGNGGCWGCAG	805r	GACTACHVGGGTATCTAATCC	17
V3-V5	357f	CCTACGGGAGGCAGCAG	926r	CCGTCAATTCMTTTRAGT	15
V4	515f	GTGCCAGCMGCCGCGGTAA	806r	GGACTACHVGGGTWTCTAAT	54
V4-V5	515f	GTGCCAGCMGCCGCGGTAA	907r	CCGTCAATTCMTTTRAGT	20
V6	967f	CAACGCGARGAACCTTACC	1061r	ACAACACGAGCTGACGAC	18

While extensive work has been published assessing best practices for characterizing free-living bacterial communities (21) or human-associated microbes as a whole (15), these findings are not directly translatable to the human vaginal microbiome for a few reasons. First, clinically important species such as Mycoplasma genitalium and Chlamydia trachomatis have an unusual pattern of substitutions in their rRNA genes, meaning that optimizing for a broad taxonomic range might have the unwanted effect of missing these species. Even more importantly, the 16S rRNA gene is generally regarded to provide taxonomic resolution only down to the genus level (22). However, for the human vaginal microbiome, distinguishing between different *Lactobacillus* species is crucial, since, e.g., Lactobacillus crispatus often plays a protective role not exerted by Lactobacillus iners (5, 7, 23).

One way to bypass the tradeoffs involved in selecting a PCR primer set is to perform full metagenomic shotgun sequencing. This approach presents several advantages and some serious challenges. Among the advantages of metagenomics is the possibility of going deeper than species-level classification, including identifying strains and specific genes. Recent work applying metagenomics to a large set of vaginal samples has identified extensive intraspecies variation in several important taxa, such as various *Lactobacillus* species, Gardnerella vaginalis, and Atopobium vaginae (24). It is also known that the degree of stability of the vaginal microbiome can be quite different between individuals (25). This sum of intraspecies variation and variable stability brings the necessity of subspecies resolution to explain why certain microbiomes are more resilient than others.

While all of the methods described above can broadly assess a wide range of taxa, they are only semiquantitative and may introduce different biases at the library preparation and bioinformatic steps. To systematically assess the effect of different variable regions, different bioinformatic approaches, and different taxonomic annotation pipelines on the observed microbial profile of human vaginal samples, we have attempted to identify all primer pairs used in published human vaginal microbiome studies in the past decade. Each of these primers was assessed *in silico* for taxonomic coverage and annotation accuracy. Different annotation schemes were used for each primer pair. The pairs with the best performance were taken into the lab and used to amplify the same set of samples. Furthermore, shotgun metagenomic sequencing was applied to each of these samples as well. This way, we can directly compare the results between primer sets and sequencing strategies.

The gold standard for quantifying specific organisms is still qPCR, a fully quantitative method. Here, we performed qPCR on three key vaginal taxa (Lactobacillus crispatus, Lactobacillus iners, and Gardnerella vaginalis) to provide a ground truth against which each of the other methods could be assessed. The results described here can guide the implementation of future vaginal microbiome studies and provide valuable information for the comparison of previous studies which have used diverging methods. A summary of all parameters assessed is presented in Table 2.

**TABLE 2:** Summary of the analyses presented in this work, including parameters varied and where to find the relevant results

Data type	Goal of analysis	Parameters assessed	Parameters kept constant	Ideal scenario	Figure(s) or table(s) presenting results
*In silico* amplicons	Assess the percentage of vaginotropic species captured by different common primer pairs	Primer pairs 27f-338r, 27f-515r, 27f-534r, 319f-806r, 341f-805r, 357f-926r, 515f-806r, 515f-907r, 967f-1061r	No errors were simulated	The ideal primer set would give 100% coverage of all genera in the database	Fig. 1, Table 2, Table 3, Table S3
*In silico* amplicons	Assess how often amplicons can be annotated to species level	Primer pairs 27f-338r, 27f-515r, 27f-534r, 319f-806r, 341f-805r, 357f-926r, 515f-806r, 515f-907r, 967f-1061r; directly mapping amplicons or using the DADA2 classifier	No errors were simulated; Only the SILVA128 database was assessed	The ideal primer set would give 100% correct species-level annotation regardless of the method used	Fig. 2
Amplicons	Assess the reproducibility of PCR triplicates	Three primer pairs (27f-515r, 27f-534r, 341f-805r) were each used in triplicate to amplify 8 different pools of samples	PCR parameters were not varied	Each triplicate would perfectly align, as well as triplicates from different primer pairs	Fig. 3a
Amplicons	Evaluate the possibility of reducing PCR biases by using a single-step PCR amplification	2-step (20 + 10 cycles) vs. 1-step (25 cycles) of amplification	PCR parameters for the first PCR are identical to the ones for the 1-step process; cleaning procedures are identical	Reducing PCR cycles would be cost effective and reduce PCR artifacts	Fig. 3b
Amplicons	Assess whether reads covering the V1-V3 regions can be merged	Total no. of reads merged and taxonomic bias in merging	Merging and taxonomic annotation procedures	Reads would be merged at a high rate and with no taxonomic bias	Fig. 3c
Amplicons	Assess the effects of the read processing strategy used on the estimated alpha-diversity	Clustering at 97%, DADA2 error correction and Unoise error correction	Read trimming and merging/concatenation were not varied	Each procedure would generate a comparable alpha-diversity and cluster size profile regardless of the parameters used	Fig. S1
Amplicons	Assess the effects of different annotation strategies on the perceived taxonomic profile	Mapping vs DADA2 classifier; SILVA, RDP, and GTDB databases	Mapping vs. DADA2 classifier was assessed against the SILVA database	Each procedure would generate a comparable taxonomic profile, which would also agree with the qPCR results	Fig. 4. Fig. S2
Shotgun sequencing	Assess the effect of different human DNA removal strategies on the rate of microbial and human reads retained	BMTagger, BBMap, Kraken in quick and sensitive modes, Bowtie2 in quick mode	Bowtie2 in sensitive mode was used as the gold standard of comparison	All microbial reads would be kept, while all human reads would be discarded	Fig. 5
Shotgun sequencing	Assess the taxonomic bias of human read removal	BMTagger, BBMap, Kraken in quick and sensitive modes, Bowtie2 in quick mode	Bowtie2 in sensitive mode was used as the gold standard of comparison	The microbial reads wrongly discarded would be evenly distributed across the microbial tree of life	No differences found
Shotgun sequencing	Assess the effect of different taxonomic annotation strategies on the perceived microbial profile	Metaphlan, Metalign, Kraken2 against their standard microbial database, Kraken2 against the OptiVag database and VIRGO	Parameters were not varied within each classifier	Each procedure would generate a comparable taxonomic profile, which would also agree with the qPCR results	Fig. 6

## RESULTS AND DISCUSSION

### Coverage of each primer.

To assess how well each primer sequence or primer combination covers potential vaginal taxa, all sequences matching each primer or primer combination were extracted from the database with regular expressions allowing only exact matches to the full length of any variant of each degenerate primer. A problem for the 27f primer variants is that many sequences in the database are incomplete at their 5′ ends, which makes this assessment impossible. The same was not true at the 3′ end: the coverage for this region does not wane until after the V8 region, so it did not affect the assessment of any primers. The total coverage of each primer is depicted in Table 3, and coverage for primer pairs is shown in Table 4. Pair 967-1061 performed much more poorly than the remainder, with the exception of the 27f primers, which could not be properly assessed.

**TABLE 3:** Coverage of each primer assessed individually

Primer	No. of counts	% coverage
27f, simple	249	26.8
27f, pool	297	31.9
319f	896	96.3
338r	909	97.7
357f	892	95.9
515f	898	96.6
534f	896	96.3
806r	848	91.2
907/926r	842	90.5
967f	670	72.0
1061r	55	5.9

**TABLE 4:** Coverage of primers in relevant pairs

Primer pair	Approach^*a*^	Proportion (%) of database matched
27f-338r	Pessimistic	26.1
27f-338r	Optimistic	95.7
27pool-515r	Pessimistic	30.9
27fpool-515r	Optimistic	96.6
27f-534r	Pessimistic	25.6
27f-534r	Optimistic	96.3
27pool-534r	Pessimistic	25.6
27pool-534r	Optimistic	96.3
319f-806r	NA	87.8
341f-805r	NA	88.5
357f-926r	NA	87.6
515f-806r	NA	88.9
515f-907r	NA	88.1
967f-1061r	NA	67.3

a NA, not applicable.

10.1128/mSphere.00448-20.1 FIG S1 Effect of error correction/clustering strategy on the estimated alpha-diversity of samples based on different metrics. Simpson’s and Shannon’s diversity scores are calculated either on the full data set or on the data set with exclusion of low-abundance ASV/OTU. Chao1 and ACE richness metrics should be calculated only on the original data set and are therefore presented only in this way. All observed effects are much larger for concatenated reads than for merged reads. Download FIG S1, PDF file, 0.04 MB. Copyright © 2020 Hugerth et al. 2020 Hugerth et al. This content is distributed under the terms of the Creative Commons Attribution 4.0 International license.

10.1128/mSphere.00448-20.2 FIG S2 Taxonomic profile of each amplicon sample, with different primer sets and different annotation databases. The reproducibility within triplicates is very high. There is good agreement between SILVA and RDP, but GTDB assigns a very large fraction of reads to *Bifidobacterium*. Download FIG S2, PDF file, 0.1 MB. Copyright © 2020 Hugerth et al. 2020 Hugerth et al. This content is distributed under the terms of the Creative Commons Attribution 4.0 International license.

10.1128/mSphere.00448-20.6 TABLE S3 Coverage of each vaginotropic genus by each primer pair combination (in percentage points). Download Table S3, CSV file, 0.01 MB. Copyright © 2020 Hugerth et al. 2020 Hugerth et al. This content is distributed under the terms of the Creative Commons Attribution 4.0 International license.

**FIG 1:**
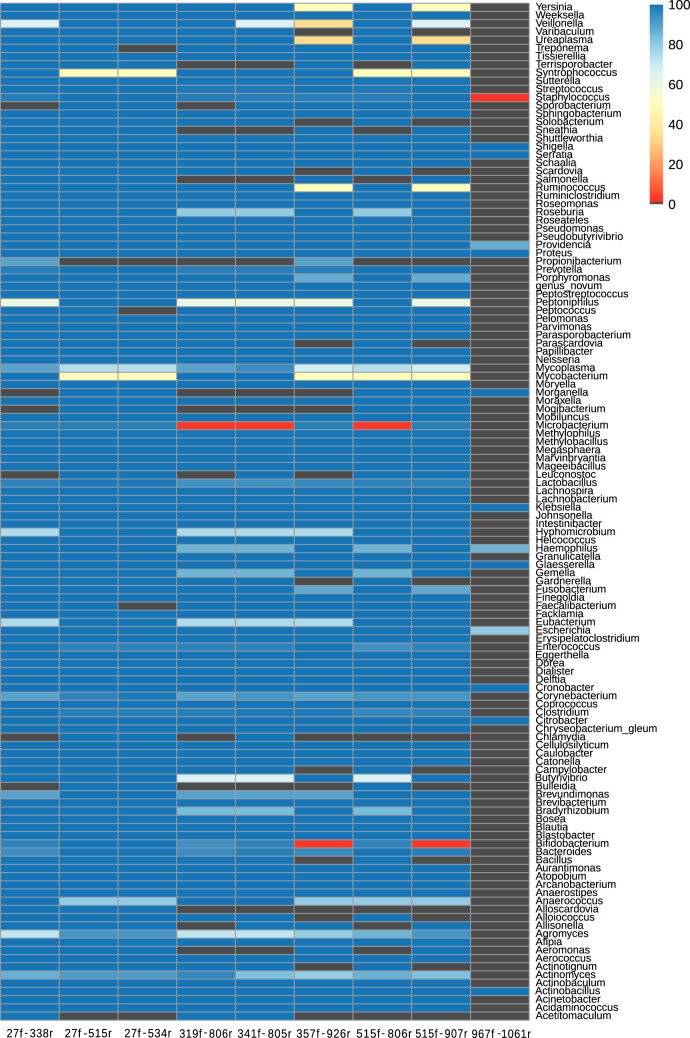
Heat map of taxon coverage at the genus level for commonly used primer pairs. Each column represents a primer pair, and each row depicts a vaginotropic genus. The percentage of sequences in each genus covered by each primer is indicated through a color scale. Most previously published work uses 16S primers with good coverage, but a few genera remain a problem, such as *Chlamydia* and *Sneathia*.

In addition to covering a large percentage of all sequences, it is important that primers avoid taxonomic bias. The taxonomic coverage of each primer pair is depicted in Table S3. Three of the genera that are mostly missed are *Propionibacterium*, *Chlamydia*, and *Mycoplasma*. *Propionibacterium* is well covered by 341f-805r and possibly 27f-338r. These same pairs perform well with *Mycoplasma*, but only the former also covers *Chlamydia*. To add *Chlamydia* coverage to the 27f pool, one extra degeneracy has to be added to the reverse primer, making it either 515r 6× 5′-GTGBCAGCMGCCGCGGTAA-3′ + 5′-GTGCCAGCAGCTGCGGTAA-3′ or 534r 5′-GTGCCAGCAGCYGCGGTAA-3′. Figure 1 shows a heat map with the taxonomic coverage of each primer pair, assuming a match of the 27f primers, for which an assessment was impossible.

Importantly, primer amplification bias goes beyond entirely missing certain clades. G+C-rich templates might perform differently than those rich in A+T (26), and taxa with longer variants might not be detected as efficiently as others with a shorter variable region (27). These biases are compounded by the exponential nature of PCR amplification. A single copy amplified with an efficiency of 1.9× per cycle will, after 30 cycles, appear to be 5 times less abundant than one amplified at perfect efficiency.

### Taxonomic annotation strategies.

**FIG 2:**
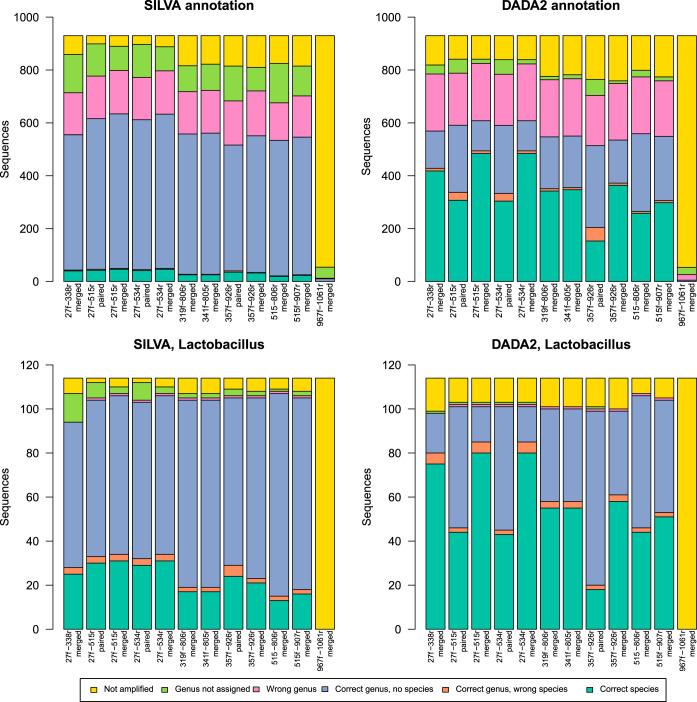
Bar plots showing the taxonomic classification accuracy of each primer pair under two classification schemes. DADA2 taxonomic annotation gives higher taxonomic resolution than mapping to a comparable database, both in general and for *Lactobacillus* in particular. The entire OptiVag database was extracted *in silico* with each of the candidate primer sets, without errors. (Top left) The complete database, annotated by mapping; (top right) the complete database, annotated with DADA2’s algorithm; (bottom left) same as panel a but focusing only on *Lacobacillus*; (bottom right) same as panel b but focusing only on *Lactobacillus*.

Even when provided with a primer pair that is potentially informative, researchers must use appropriate bioinformatic pipelines to retrieve this information. At this step, we assume that we have perfect error correction capability and do not attempt to simulate PCR and sequencing errors. For long amplicons, where merging of forward and reverse reads might not be possible, we present results for both merged and unmerged reads. Figure 2 (top panels) presents the taxonomic accuracy for each primer pair and taxonomic annotation strategy for the full set of vaginal taxa. V1-V2 and V1-V3 perform better for the vaginal microbiome than other regions, provided that they are merged, since processing reads separately entails a loss of precision and accuracy as large as a switch to a different region. Species-level accuracy is particularly critical for genus *Lactobacillus*, since, e.g., *L. iners* is associated with a very different outcome for the host subject than L. crispatus (5, 23). Figure 2 (bottom panels) presents bar plots of taxonomic accuracy for the 114 *Lactobacillus* species included in our study. The trends observed are very similar to the ones observed for the full vaginal database.

### Amplicon sequencing.

To assess the accuracy of these algorithms, 8 pools of vaginal swabs (coming from 4 consecutive days of sampling from a single individual each) were amplified using either the V1-V3 or the V3-V4 region. For the V1-V3 region, two primer pairs were assessed: 27f-534r has the potential to amplify Chlamydia trachomatis, which is lacking from most other primer pairs. However, its length could create other issues, which is why 27f-515r was also assessed. The V3-V4 regions was amplified by the primer pair 341f-805r. The results observed for this pair can be naturally extended to the also popular pairs V3V4 341f-806r and V4 515f-806r.

**FIG 3:**
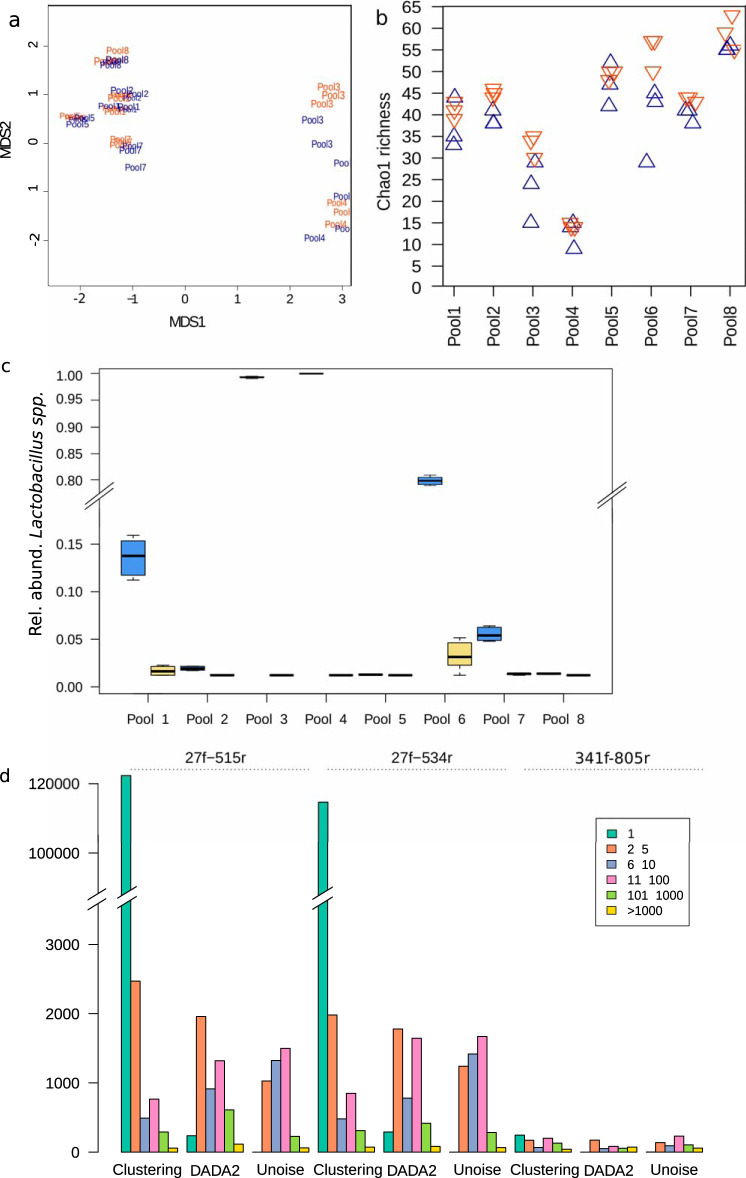
Effect of various analysis parameters on alpha- and beta-diversity of real amplicons. Orange, V3-V4, 2-step PCR; blue, V3-V4, 1-step PCR; green, V1-V3–515r; gray, V1-V3–534r. (a) Nonmetric multidimensional scaling of the 8 pools, processed in triplicate with V3-V4 primers, shows good replicability within triplicates and regardless of PCR set-up (single-step versus nested reactions). (b) Chao1 richness estimate for each of the samples in panel a. The 1-step PCR approach generally yields a lower richness estimate but has slightly higher variability within triplicates. (c) Box plots depicting the estimated relative abundance of *Lactobacillus* spp. in each sample when the reads were merged or simply concatenated. There is a disproportional loss of *Lactobacillus* spp. upon attempting to merge long amplicons. (d) Bar plots showing the number of ASV or operational taxonomic units (OTU) of different cluster size classes obtained with each primer pair and error correction or clustering method. The effect of these choices on alpha-diversity estimates can be seen in Fig. S1.

For the V3-V4 region, two experimental approaches were compared, using either a single PCR (which both amplifies this region and barcodes it), or two consecutive PCRs (one for amplification and one for barcoding). The one-step PCR approach is more cost-effective, since a single cleaning step is necessary, and minimizes the risk of cross-contamination between wells, since at no point are there samples amplified but not barcoded. However, the long PCR primers can be challenging to obtain, and reaction conditions are also more delicate. Here, both approaches performed very similarly (Fig. 3a), but in some replicates there is a difference in richness (Fig. 3b). These results mean that either the 2-step approach produces more artifacts or the 1-step approach did not capture the full richness of the sample due to worse PCR performance for the long primers. Since the triplicates for the two-step approach yielded more similar results, the latter is the more likely explanation. However, it is also worth considering that while the negative extraction controls for the 1-step approach yielded a total of 3 16S reads post-quality control (QC), the 2-step approach had 2,044 reads, highlighting the risk of working with amplified but not barcoded molecules, especially in a high-throughput setting.

The V1-V3 amplicons are too long for current paired-end 300-bp approaches to accurately bridge the space between reads. Although ca. 80% of reads in each sample could be merged (medians, 79% for 27f-5153 and 85% for 27f-534r), there is a strong taxonomic bias on the reads kept. Indeed, for pools 3 and 4, which are strongly *Lactobacillus* dominated, less than 1% of reads could be merged. Figure 3c shows the percentage of *Lactobacillus* in each sample pool when merging or concatenating (classified with the DADA2 classifier on the SILVA database). Due to this strong bias, read concatenation must be used rather than read merging. Failing to merge decreases the accuracy of this middle region, which is generally already low due to the failing accuracy of sequencing along the read length (28). This poses a challenge. To achieve species-level resolution and an accurate estimate of total species, it has been shown to be crucial to use an error correction strategy rather than a clustering one (29). However, the additional errors kept by not merging reads could potentially make error correction more error prone than simple clustering. Here, we compared two error-correcting strategies, DADA2 (30) and Unoise3 (31), as well as traditional average-linkage clustering at 97% identity.

DADA2 is optimized to correct sequencing errors and will not eliminate PCR errors, so this algorithm is recommended only in combination with a high-fidelity polymerase to avoid large numbers of false positives. Unoise3 eliminates both amplification and sequencing errors but also presents a higher risk of excluding rare but correct sequences, which generally makes Unoise3 a more conservative approach (29). Indeed, in the case of error-prone concatenated reads, DADA2 generated more low-abundance amplicon sequence variants (ASV) than Unoise (Fig. 3d). Clustering yielded even more ASV than error correction, strongly suggesting that the ASV results are more correct. These differences also affect estimates of alpha-diversity (Fig. S1). Since estimates of diversity can be both over- and underestimated due to a large added number of singletons, differences in Shannon's and inverted Simpson's diversity were not significantly different between methods. Estimates of richness, however, were all significantly different between methods (all *P* < 0.0001), with Unoise giving the lowest estimates and clustering the highest.

**FIG 4:**
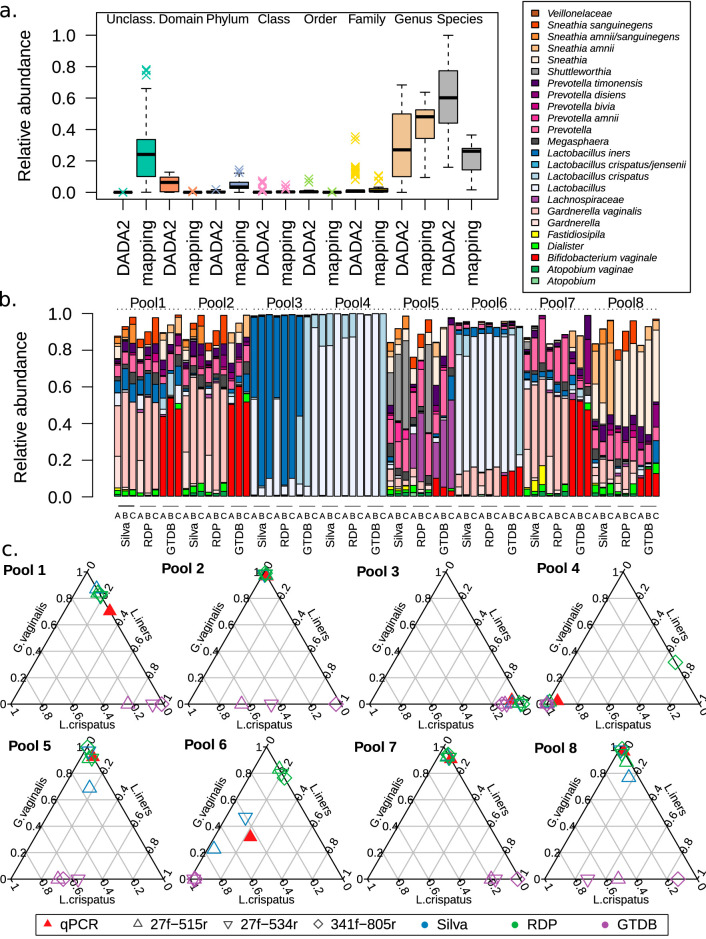
Effects of various parameters on the taxonomic annotation of real amplicons. The taxonomic accuracy of each of the 16S primer sets is good, but V1-V3–534r yields more shallow annotations. It can, however, reliably detect Chlamydia trachomatis spike-in DNA. (a) Box plots showing the depth of classification for each sequence with different classification strategies. The DADA2 classifier yielded higher taxonomic resolution thatn simply mapping, regardless of the database used. (b) Taxonomy bar plots for each of the pools, processed with 2-step PCR with each of the primer sets and with each of the databases SILVA, RDP, and GTDB. An average for each triplicate is shown. Each technical replicate can be seen in Fig. S2. Only ASV with >10 counts are included in this figure. (c) Same samples as in panel a, compared to qPCR results for Lactobacillus iners, Lactobacillus crispatus, and Gardnerella vaginalis. For each sample, the sum of these three taxa was normalized to 1, to make them comparable to the qPCR results in the triaxial plot.

DADA2 taxonomic assignment had a higher rate of reads assigned at the species level than the mapping strategy, confirming the results in Fig. 2 (Fig. 4a). The taxonomic composition of each PCR triplicate analyzed with the best possible setup for each primer set is highly comparable (Fig. S2). The effect of the database used for annotation can be larger than that of the region used. Remarkably, the very well established SILVA and RDP databases yield very similar results (Fig. 4b). Primer pair V1-V3–534r yielded slightly worse taxonomic resolution than the other two primers analyzed. Compared to qPCR, all three approaches are extremely accurate, regardless of the database used (Fig. 4c).

Despite its somewhat lower taxonomic resolution with the read lengths obtained, primer pair V1-V3–534r is the only one expected to amplify and detect Chlamydia trachomatis. To confirm this, a spike-in experiment was conducted (Fig. S3). The varying amount of human DNA initially found in each sample means that a spike-in of 5% of total DNA may correspond to >50% of bacterial DNA, making this analysis harder to interpret. In general, there is a good correlation between spiked-in and observed C. trachomatis.

10.1128/mSphere.00448-20.3 FIG S3 Percentage of *C. trachomatis* detected in each sample as a function of the DNA spike-in. Differences in human DNA content affect the observed bacterial counts, and for the three samples with highest DNA content (pools 3, 4, and 6), the assay quickly becomes saturated. Dashed gray lines mark 1%, 5%, and 10%, which were the proportions used for the spike-in experiment. Download FIG S3, PDF file, 0.03 MB. Copyright © 2020 Hugerth et al. 2020 Hugerth et al. This content is distributed under the terms of the Creative Commons Attribution 4.0 International license.

### *In silico* removal of human DNA from metagenomic data.

An alternative to PCR amplification is performing full shotgun metagenomic sequencing of samples. The first challenge for processing metagenomic reads derived from vaginal swabs is the large amount of human DNA in these samples. In our pools, 86 to 98% of the reads could be mapped to the human genome. While human DNA depletion can be performed *in vitro* prior to sequencing (32, 33), this depends on the storage condition of the samples and was not evaluated in this work. Instead, we focused on *in silico* removal of human reads.

**FIG 5:**
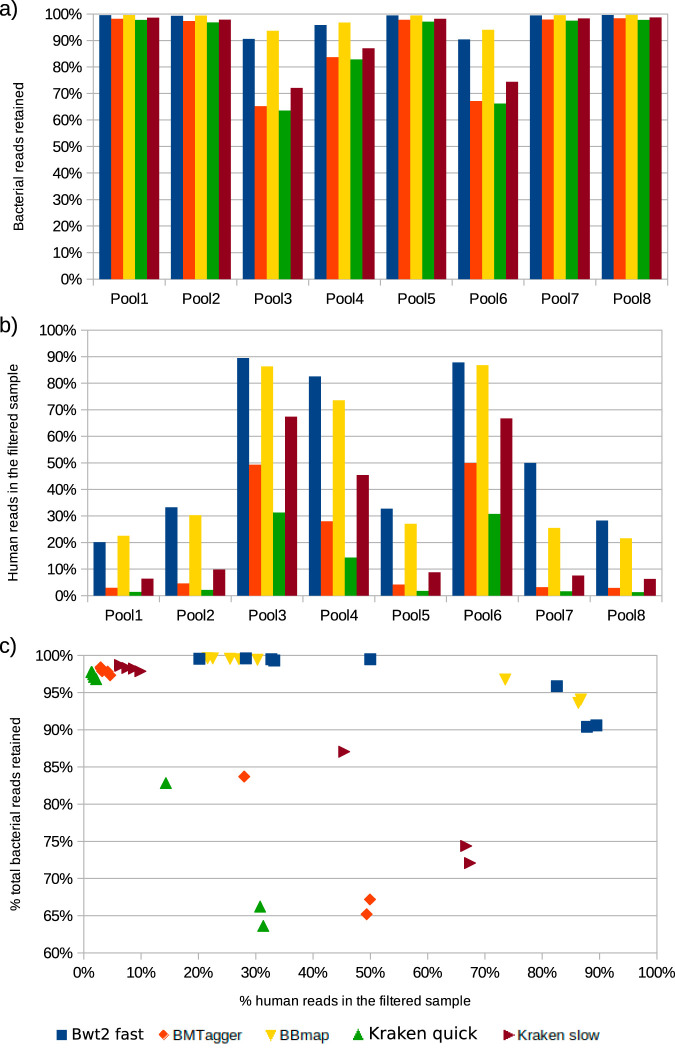
Effect of human-DNA removal strategies on the amount of bacterial and human DNA retained. The human-DNA content in the 8 pools varied from 86 to 98% of the total DNA content. Different DNA removal methods have various amounts of human DNA left in the filtered sample but also retain various amounts of the original microbial pool. Kraken and BMTagger remove most human DNA but also the most microbial reads. (a) Percentage of human reads in each sample before and after each human removal strategy. (b) Percentage of the original pool of microbial reads kept in each sample after each human removal strategy. (c) The two measurements in a and b are combined into a scatterplot to give an overview of the performance of each tool.

Removal of reads of human origin is a conceptually straightforward process consisting of mapping reads to a reference genome. However, two critical factors can affect the outcome: the mapping algorithm and the masking applied to the reference genome to hide regions exhibiting homology to *Bacteria* and *Fungi*. Strict mapping is time and memory intensive. A looser mapping is less resource intensive but might remove more bacterial reads or retain more human reads, depending on how strictly the reference is masked. Many mappers provide a preformatted human genome reference for host removal. Here, we tested three of them: BMTagger, BBmap, and Kraken2, the latter in both “quick” and “standard” modes. We also ran Bowtie2 in –fastlocal settings to contrast it with the –very-sensitive-local settings that we used as the gold standard for this analysis. The percentage of human DNA left in each sample after human DNA removal with the different techniques is depicted in Fig. 5a. The percentage of bacterial DNA kept, from the initial bacterial pool, is shown in Fig. 5b. These two quality scores are combined in Fig. 5c, where the optimal method would place all samples in the upper left corner. BBMap and Bowtie2 retained the most human DNA but also the most bacterial reads. Conversely, BMTagger and Kraken2 removed the most human reads, at the expense of also decreasing the microbial pool. Based on the results above, Kraken2, in quick mode, was chosen for downstream analysis.

Interestingly, all tools followed the same general trends, removing more bacterial reads and also retaining more human reads in samples with an initially very high (>95%) human DNA content. Of notice, these three samples (pools 3, 4, and 6) also have the highest *Lactobacillus* counts. To assess whether removal of human content causes selective removal of specific bacterial taxa, we also attempted to assign taxonomy to these putative human reads (detected with Bowtie2 in very-sensitive-local mode). For each sample pool, >98.4% of putative human reads could not be assigned to any bacterial genome, strongly suggesting that these are indeed eukaryotic reads. About two-thirds of the 1.5% of reads are classified as *Zoebellia*, a genus of marine *Flavobacteriaceae* not known to infect humans. The remaining third is chiefly assigned to Chlamydia psittaci and Chlamydia abortus. While we have not evaluated the read alignments in detail, we speculate that the reference genome of these intracellular parasites may contain small amounts of sequences of human origin, generating this misleading assignment. Therefore, the larger amount of human DNA observed in *Lactobacillus-*rich samples is likely to be true human DNA, connected to the shedding of glycogen-rich epithelial cells that feeds the *Lactobacillus* community.

### Taxonomic annotation of metagenomic data.

**FIG 6:**
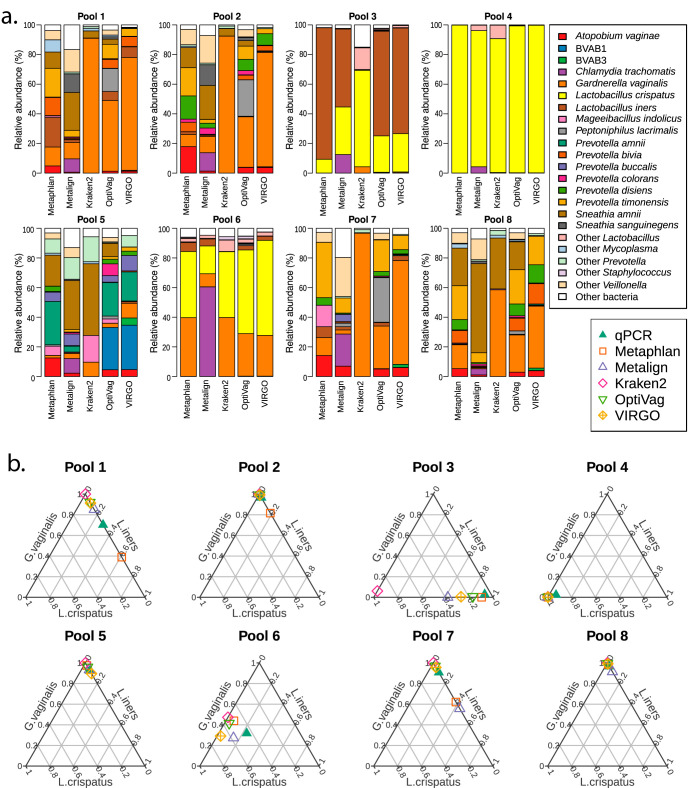
Effect of taxonomy assignment strategy on the perceived taxonomic profile of each sample. Assigning taxonomy to shotgun metagenomic reads with various tools yields somewhat different community profiles. (a) Taxonomy for each pool assigned with Metaphlan, Metalign, or Kraken2 to its complete microbial database, Kraken2 to the OptiVag database, or VIRGO. (b) Same samples as in panel a, compared to qPCR results for Lactobacillus iners, Lactobacillus crispatus, and Gardnerella vaginalis. For each sample, the sum of these three taxa was normalized to 1, to make them comparable to the qPCR results in the triaxial plot. (c) Manhattan distance between each sample and method and its corresponding qPCR profile. In this three-dimensional structure, the Manhattan distance is strictly limited between 0 (identical profiles) and 3 (maximum distance for each of the three species considered).

Five approaches were assessed for taxonomic assignment on these data: a general marker gene-based approach (MetaPhlAn2), a marker gene-based approach built from a curated set of vaginal bacteria (VIRGO), a k-mer-based approach with a broad taxonomic database (Kraken2; see Materials and Methods for details), a k-mer-based approach with a vaginal-only database (Kraken2), and a novel prefiltering and alignment tool (Metalign). The taxonomic profile inferred by each method for each pool is depicted in Fig. 6a. Metalign stands out in identifying Chlamydia trachomatis in almost every pool, as well as a higher frequency of detection of *Veillonella* spp. and *Prevotella* spp. The standard Kraken2 database failed to identify *L. iners*, despite this species being present in the database. Kraken2 with OptiVagDB, Metaphlan, and VIRGO tended to present similar results, with a few notable differences. First, the clade called BVAB3 in VIRGO takes its current name Mageeibacillus indolicus in the other two references. Metaphlan fails to identify BVAB1, perhaps because this genome is still not in NCBI’s RefSeq database. OptiVag is alone in identifying significant amounts of *Peptoniphilus* in three of the *Gardnerella*-dominated samples. This clade has been identified in women with bacterial vaginosis (34) but is generally not considered a key taxon for this condition. Finally, VIRGO stands out in not identifying any *Sneathia* organisms, even in samples where all other methods are in agreement.

Comparison to qPCR showed that none of the shotgun methods was as accurate as the PCR-based methods (Fig. 6b; contrast to Fig. 4b). Still, when each pool is considered, VIRGO and OptiVag performed better than the other methods (Fig. 4c). It is possible that assessing taxonomy after assembly would yield more accurate results (35), but this was not possible with the current sampling depth. Still, this could be a valid alternative for samples sequenced more deeply, or for a different experimental design, e.g., a time series from the same woman, which would enable coassembly across closely related samples.

### Conclusions.

None of the methods assessed here is superior in all respects. With regard to amplicons, V3-V4 yielded the most plausible alpha-diversity estimates and had very good taxonomic coverage. However, much of the existing literature is based on region V1-V3 (14–16). The major drawback of 16S amplicons is their failure to detect eukaryotic taxa such as *Candida* spp. and Trichomonas vaginalis. An ITS (internal transcribed spacer)-based amplicon approach could selectively amplify fungi without amplifying human DNA (36), but it would miss the pathogenic parabasalid T. vaginalis. Therefore, no simple combination of one or two primer sets can accurately profile all relevant taxa in the human vaginal environment.

To overcome the limitations imposed by primer selection, shotgun metagenomic sequencing presents an interesting alternative, since it is not *a priori* bound by phylogeny. Its cost, which used to be prohibitive, is now low enough to compete with a multiprimer PCR-based approach. In addition to taxonomic classification, shotgun data allow researchers to assess the functional gene content of a sample and, given enough sequencing depth, assemble draft genomes of strains of interest.

The main practical obstacle to a broader application of shotgun metagenomics in the field of obstetrics and gynecology is the large amount of human DNA in vaginal swabs, but this can potentially be bypassed, either with molecular biology techniques or a combination of deep sequencing and *in silico* human DNA removal. The bioinformatic skill set and computational requirements necessary to handle this type of data are also significantly larger than those needed for marker gene (16S) analyses.

Comparing data sets derived from amplicon or shotgun sequencing also requires an understanding of the specific biases in each of these technologies. Despite using different primer sets and enzymes, it is not entirely unexpected that the PCR-based data have better agreement with the qPCR data, since these share many common biases, such as copy number variations. The linear amplification strategy used with DNBSeq (37) is potentially less biased than PCR-based strategies, but these claims have not yet been supported by independent research groups. The role of GC bias, which is significant for most other massively parallel sequencing technologies (26), is also currently unknown for this technology.

Here, we present a thorough comparison of multiple methods available for the survey of the vaginal microbiota. Since none of the methods is universally optimal, it is still up to each research center to select the appropriate method for their specific research question. While this will necessarily limit comparability between studies, acknowledging the strengths and weaknesses of each method is already a substantial improvement to the current state of the field.

## MATERIALS AND METHODS

### Construction of the databases.

To create a corresponding shotgun database, we started from the list of vaginotropic species published by Diop et al. (38). In addition to these previously published results, a data set of 480 vaginal swabs collected throughout the menstrual cycle of a healthy Danish cohort (M. C. Krog et al., submitted for publication) and sequenced by CoreBiome (St. Paul, MN, USA) using BoosterShot technology was used. For every bacterial species identified in the data set and not present in the Diop database, manual searches of PubMed and NucCore were done, and the species was kept if it had been previously identified in the human urogenital tract. Eukaryotic species were added by searching NucCore with the search key “((vagina[All Fields] AND “Eukaryota”[Organism]) NOT “Metazoa”[Organism]) NOT “Viridiplantae”[Organism] AND (biomol_genomic[PROP] AND refseq[filter]).” Finally, a free-text search for “BVAB” retrieved metagenome-associated genomes representative of the bacterial vaginosis-associated *Clostridiales* group. The resulting list of taxa is available in Table S1. When a taxon could not be programmatically included in the database, manual searches against NCBI’s Taxonomy database were used to verify whether the taxon name had been updated. Not all taxa could be retrieved as full genomes, as some are present in the databases only as single genes; these taxa are missing from the current version of the database. The resulting database (v0.1) and the scripts used for producing a genome database based on a taxon list are available at https://github.com/ctmrbio/optivag/tree/master/database.

10.1128/mSphere.00448-20.4 TABLE S1 Species including in the OptiVagDB, including the source of their description as vaginotropic. Download Table S1, CSV file, 0.08 MB. Copyright © 2020 Hugerth et al. 2020 Hugerth et al. This content is distributed under the terms of the Creative Commons Attribution 4.0 International license.

### Simulated amplicons.

Amplicons were extracted from the 16S rRNA gene database based on exact matches to the primers. For amplicons starting at the 27f position, which is often not included in the reference sequence due to its location, two alternative approaches were compared. The pessimistic approach extracts only sequences containing the primer regions, while the optimistic assumes that all sequences lacking the 5′ end would be amplified by the 27f primer. The truth is likely somewhere between these extremes.

*In silico* reads were extracted from the OptiVagDB v0.1 for each primer pair, using a read length of 250 bp. While it is possible to sequence longer fragments with the commercial kits available today, this is a realistic read length after trimming primer pairs and low-quality base pairs. We did not simulate PCR and sequencing errors for these reads, since the goal of this step was to assess the performance of primers under ideal conditions. For amplicons <500 bp long, the resulting reads were merged; otherwise, they were treated independently. When the resulting amplicon length was very close to 500 bp, both approaches were considered, since the ability to merge reads becomes dependent on the accuracy of the sequencer used.

### Sample collection.

Women were recruited by advertisements in student magazines, university notice boards, and social media and were included between September 2017 and January 2018 at Rigshospitalet, Copenhagen, Denmark. The women were provided with self-collection kits and received instructions for vaginal swab collection. In short, they were instructed to separate the labia major with one hand (in order to reduce the risk of contamination with microbiota from external genitals), insert a swab (FLOQSwabs [CP520CS01; Copan Flock Technologies, Brescia, Italy]) into the vagina with the other hand, and rotate it for 10 to 15 s before placing the swab in the provided collection tube (FluidX tube [65-7534; Brooks Life Sciences, Chelmsford, MA, USA] containing 0.8 ml DNA/RNA-shield [R1100-250; Zymo Research, Irvine, CA, USA]) and breaking off the handle. Samples were kept at room temperature for up to 2 weeks and then at −20°C for up to 4 weeks before being transferred to −80°C. All participants gave oral and written consent to participate in the study and were remunerated with 3,000 Danish kroner (DKK) after completing sample collection. All data were collected and managed using REDCap electronic data capture tools (39), hosted at the Capital Region of Denmark. The study was approved by The Regional Committee on Health Research Ethics (H-17017580) and the Data Protection Agency in the Capital Region of Denmark (2012-58-0004).

### DNA extraction.

DNA extraction was performed with the Quick-DNA Magbead Plus kit (D4082; Zymo Research, Irvine, CA, USA), according to the manufacturer’s instructions with few modifications. Prior to extraction, the samples were subjected to bead beating for 1 min at 1,600 rpm using ZR Bashing Bead lysis matrix (S6012; Zymo Research, Irvine, CA, USA). After bead beating, samples were treated with a lysozyme solution 37°C for 60 min (lysozyme recipe: 20 mM Tris-Cl, pH 8; 2 mM sodium EDTA [Tris-EDTA; Sigma-Aldrich, catalog no. T9285]; lysozyme [Sigma-Aldrich, catalog no. L6876-100G] to 100 mg/ml) and proteinase K at 55°C for 30 min (20 mg/ml, part of the extraction kit), previously to DNA cleanup using a Freedom EVO robot (Tecan, Männendorf, Switzerland). Eight sample pools were created for this study, consisting of 4 consecutive daily vaginal swabs from each of 8 individuals from a cohort of healthy young women. All eight sample pools were used for each of the experimental approaches attempted.

### Sequence amplification, sequencing, and error correction.

The following PCR set-ups were used: (i) one-step PCR amplification of the V3-V4 region, (ii) two-step PCR amplification of the V3-V4 region, (iii) two-step PCR amplification of the V1-V3 region using reverse primer 515r, and (iv) two-step PCR amplification of the V1-V3 region using reverse primer 534r. The same settings were used for an experiment with a *Chlamydia* DNA spike-in (gblocks gene fragment; Integrated DNA Technologies, Coralville, IA, USA). DNA was spiked in at 1%, 5%, or 10%.

The primer sequences and specific PCR conditions are described in Table S2. All PCRs were performed in 50-μl reaction mixtures using Phusion Hot Start II high-fidelity PCR master mix (F-565L; Thermo Fisher Scientific, MA, USA). The 1-step PCR included 1.5 μl of dimethyl sulfoxide (DMSO). All PCR products were purified with Agencourt AMPure XP beads (A63881; Beckman Coulter, Brea, CA, USA). For the two-step reactions, the purified sample was used as the template for barcoding with Nextera XT index kit v2 (FC-131-1002; Illumina, Inc., San Diego, CA, USA). The finished libraries were normalized to 4 nM, pooled, and sequenced in a MiSeq system using V3 chemistry (Illumina, Inc.).

10.1128/mSphere.00448-20.5 TABLE S2 PCR conditions for all reactions described in this work. Download Table S2, CSV file, 0.00 MB. Copyright © 2020 Hugerth et al. 2020 Hugerth et al. This content is distributed under the terms of the Creative Commons Attribution 4.0 International license.

Cutadapt (40) was used to trim primers, remove sequences not containing the expected primer pairs, and remove bases with a Phred score of <15.

Merging and error correction was performed with DADA2 (30) or Unoise (31), as described in Results. For amplicons in the V1-V3 region which were too long to be merged appropriately, concatenation of the forward and reverse reads was performed. In this case, reads were trimmed to 270 bp each. Amplicons for which at least one read did not reach 270 bp with a Phred score of >15 were discarded. Amplicons for which the expected error rate over the resulting 540 bp was >4 were discarded. The resulting concatenated products were subjected to either error correction as described above or clustering at 97% identity and chimera removal with Vsearch (41).

### Taxonomic annotation of amplicons.

Taxonomic annotation of *in silico* amplicons was performed with DADA2’s (v1.5) (30) built-in sequence classifier, based on the SILVA database (v128) (42), or by direct mapping to the SILVA v128 database. In addition to these two approaches, real amplicons were also classified with the DADA2 classifier against the RDP v16 (43) or GTDB v86 (44).

For amplicons that could not be merged, a consensus between the potentially distinct annotations of forward and reverse reads was established as follows. (i) If the two annotations were incompatible, the lowest common ancestor was kept (e.g., in cases where families agreed but genera diverged, only family-level annotation was kept). (ii) If one annotation was more detailed than the other (e.g., to genus level versus to family level) but the two annotations agreed on all levels where they overlapped, the most detailed annotation was kept. (iii) For species-level annotations where more than one species was possible, the intersection of the species suggested for each of the reads was kept (e.g., if the forward read was annotated as “Lactobacillus crispatus*/gasseri/jensenii*” and the reverse as “Lactobacillus gasseri*/jensenii/longum*,” the resulting annotation would be “Lactobacillus gasseri*/jensenii*”).

### Metagenomic shotgun sequencing.

The same eight pools were used for whole-genome library preparation. MGI FS DNA library prep kit (16×, 1000006987; MGI, Shenzhen, China) was used according to the manufacturer’s instructions, except that 50 ng of DNA was used as input instead of the suggested 200 ng. Due to the smaller amount of input DNA, instead of double bead cleanup for size selection, a single cleanup step was applied. MGI sequencing technology uses enzymatic fragmentation of DNA followed by barcoding of samples using PCR (7 PCR cycles in this study), single-strand circularization, and DNA nanoball construction. All procedures were automated using SP-960 and SP-100 robots (MGI). The sequencing step was performed in a DNBSEQ-G400 sequencer (MGI) using the high-throughput sequencing set (PE150 1000016952; MGI) with DNA libraries loaded onto to the flow cell using the DNB loader MGIDL-200 (MGI).

### Human-DNA removal.

Human reads were removed *in silico* by one of the following strategies: (i) Bowtie2 v2.3.5 (45) with the setting –fast-local; (ii) BMTagger v1.1.0 (46) mapping to the GRCh38 reference library with standard masking; (iii) BBMap v38.68 (47) against the hg19 reference library, masked as described in http://seqanswers.com/forums/showthread.php?t=42552; (iv) Kraken2 v2.0.8-beta (48) against its built-in GRCh38 human reference, setting the confidence parameter to 0.1; (v) Kraken2 with the parameters named above, adding flag –quick.

To be able to independently assess the human read removal performance of the aforementioned methods, reads were mapped to the hg19 masked reference using Bowtie2 v2.3.5 (45) with the setting –very-sensitive-local.

### Taxonomic annotation of shotgun reads.

For assigning taxonomy to the remaining microbial reads, four approaches were assessed: (i) Metaphlan2 v2.9.21 (49) with standard parameters; (ii) Kraken2 v2.0.8-beta (48) to a general database (built using –download-library flags for archaea, bacteria, viruses, fungi, and human) setting confidence to 0.5, followed by Bracken v2.0 (50) with threshold set to 1 read per million; (iii) Kraken2 with the same parameters, except for using the curated vaginal database described above; (iv) Metalign v0.9.1 (51) with length normalization.

### qPCR quantification of key taxa.

To further validate the results observed by sequencing, three key taxa, namely, Lactobacillus crispatus (VPI-3199), Lactobacillus iners (ATCC-55195), and Gardnerella vaginalis (CCUG-44120) were quantified by qPCR using LightCycler 480 (Roche, Mannheim, Germany) and a SYBR green assay from Bio-Rad (1725270; Bio-Rad, Sundbyberg, Sweden). The primer sequences and PCR conditions are described in Table S2. These primers were originally described by Zozaya-Hinchliffe et al. (52) and were further validated by Akutsu et al. (53) In the triaxial plots presented, the sum of these three taxa is normalized to 1 for each method presented, to allow a direct comparison.

### Data availability.

All sequencing data analyzed in this study are available from the European Nucleotide Archive under project number PRJEB37382. 16S reads have the identifiers ERR4704801 to ERR4704929, and shotgun reads have the identifiers ERR4705195 to ERR4705329.
