# Erratum for Hugerth et al., “Assessment of *In Vitro* and *In Silico* Protocols for Sequence-Based Characterization of the Human Vaginal Microbiome”

**DOI:** 10.1128/mSphere.01253-20

**Published:** 2020-12-23

**Authors:** Luisa W. Hugerth, Marcela Pereira, Yinghua Zha, Maike Seifert, Vilde Kaldhusdal, Fredrik Boulund, Maria C. Krog, Zahra Bashir, Marica Hamsten, Emma Fransson, Henriette Svarre Nielsen, Ina Schuppe-Koistinen, Lars Engstrand

**Affiliations:** a Centre for Translational Microbiome Research, Department of Microbiology, Tumour and Cell Biology, Science for Life Laboratory, Karolinska Institutet, Solna, Sweden; b Department of Medicine Solna, Division of Infectious Diseases, Karolinska University Hospital, Center for Molecular Medicine, Karolinska Institutet, Solna, Sweden; c The Recurrent Pregnancy Loss Unit, Capital Region of Denmark, Rigshospitalet and Hvidovre Hospital, Copenhagen, Denmark; d Department of Clinical Immunology, Copenhagen University Hospital, Copenhagen, Denmark; e Department of Obstetrics and Gynaecology, Holbæk Hospital, Holbæk, Denmark; f Department of Women’s and Children’s Health, Uppsala University, Uppsala, Sweden; g Department of Obstetrics and Gynecology, Hvidovre Hospital, Copenhagen, Denmark

## ERRATUM

Volume 5, no. 6, e00448-20, 2020, https://doi.org/10.1128/mSphere.00448-20. Page 1: This article was published on 18 November 2020 with the 11th author’s name misspelled. The byline was updated in the current version, posted on 16 December 2020.

