# Supplementary figures and images for: Assessment of In Vitro and In Silico Protocols for Sequence-Based Characterization of the Human Vaginal Microbiome
Source: mSphere. 2020 Nov 18;5(6):e00448-20. doi: 10.1128/mSphere.00448-20 (PMC7677004; doi:10.1128/mSphere.00448-20)

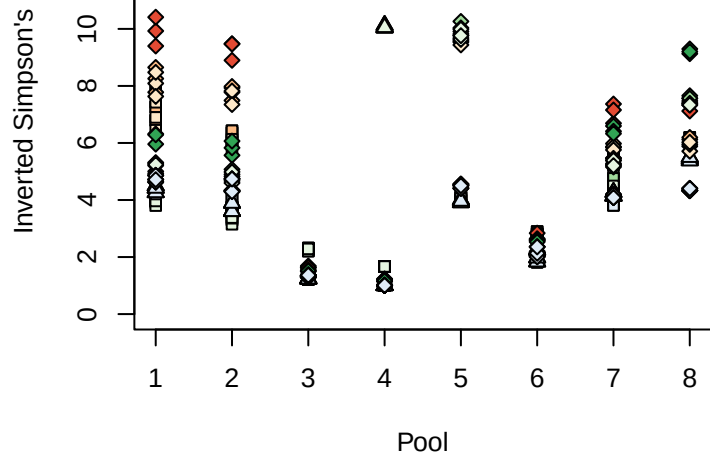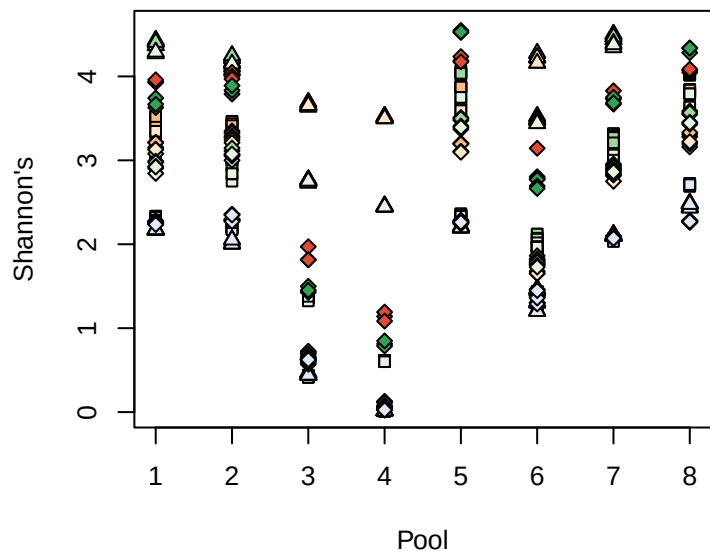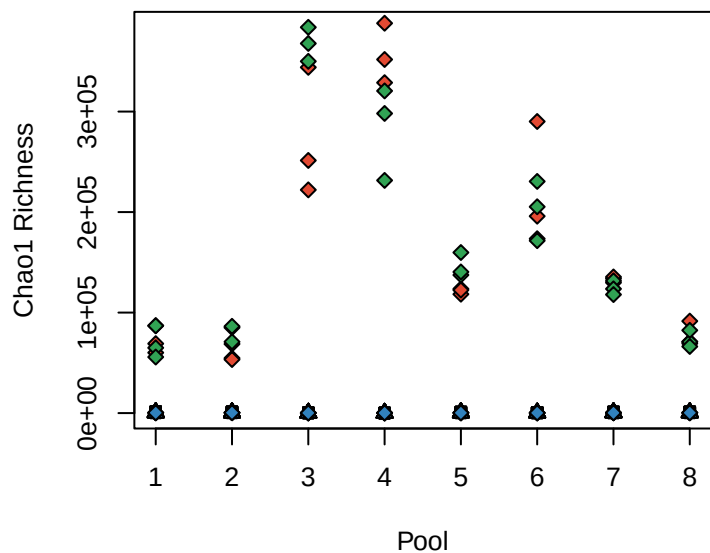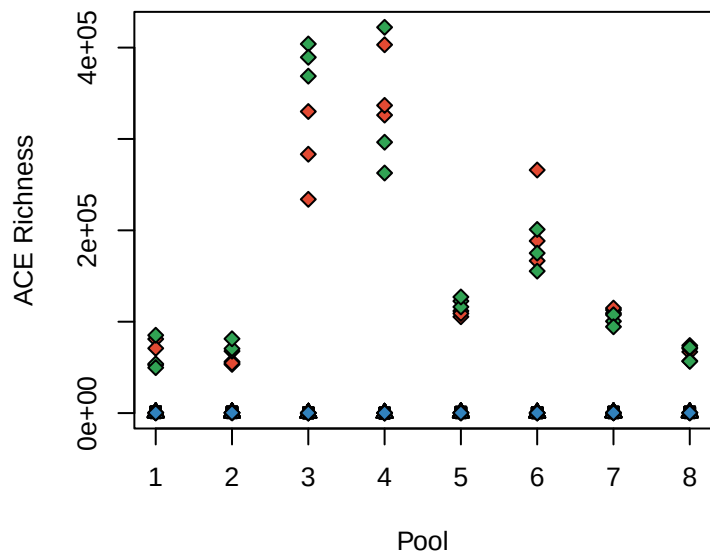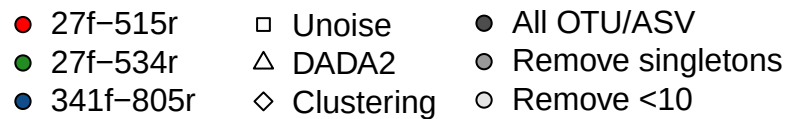

Supplement: FIG S1 [file mSphere.00448-20-sf001.pdf]

Silva 128

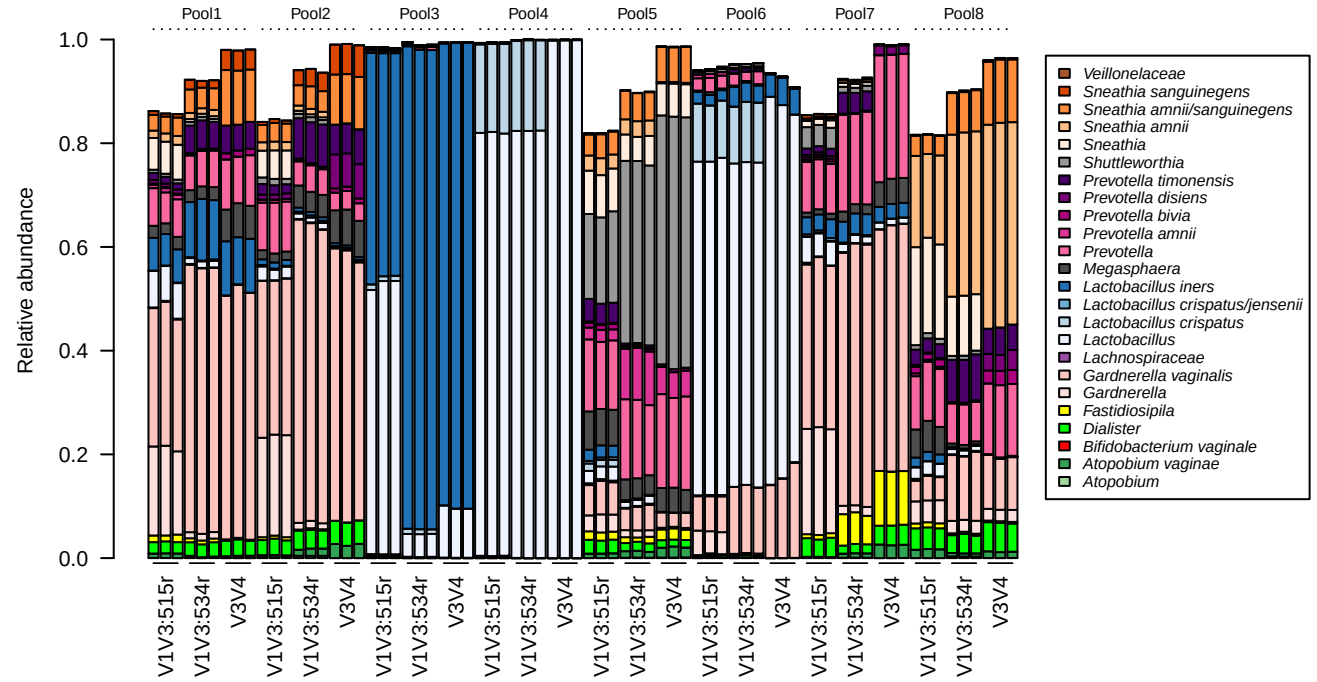

RDP 16

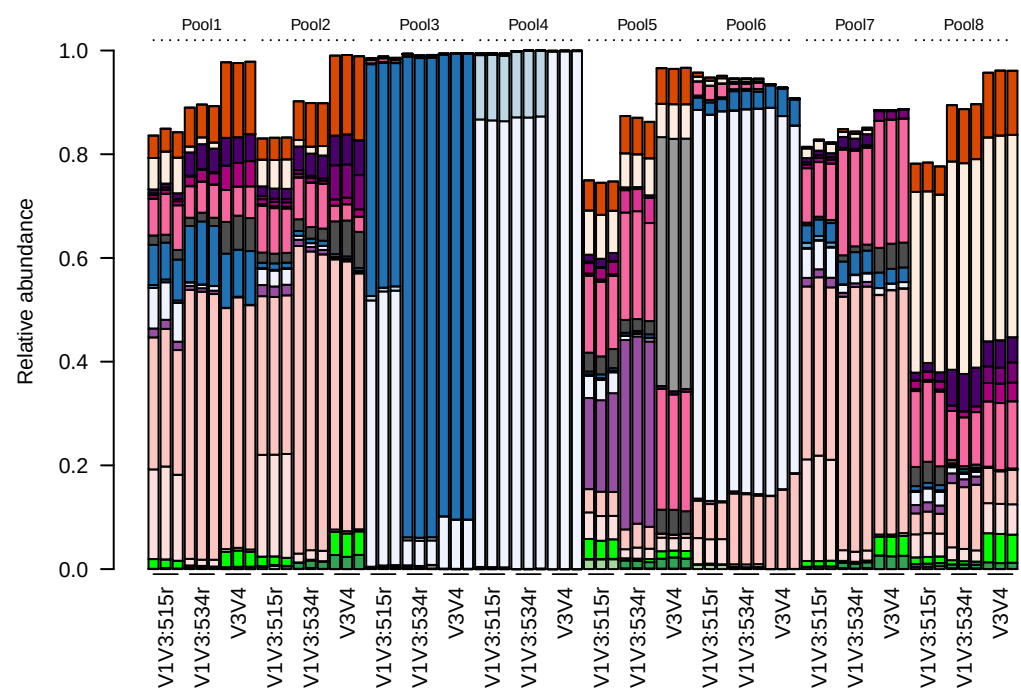

GTDB 86

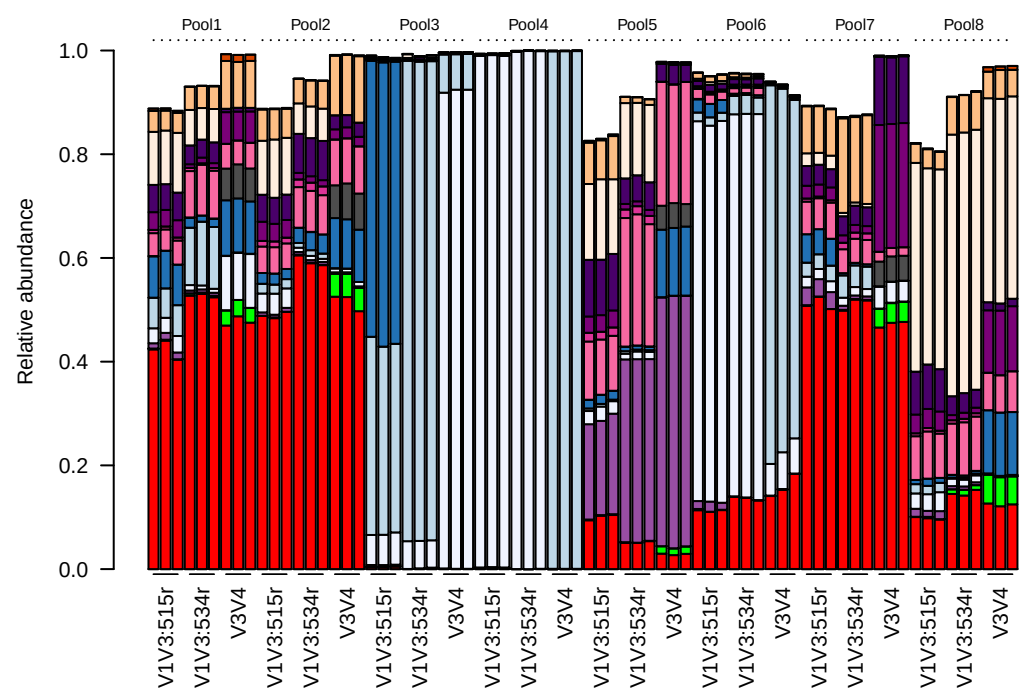

Supplement: FIG S2 [file mSphere.00448-20-sf002.pdf]

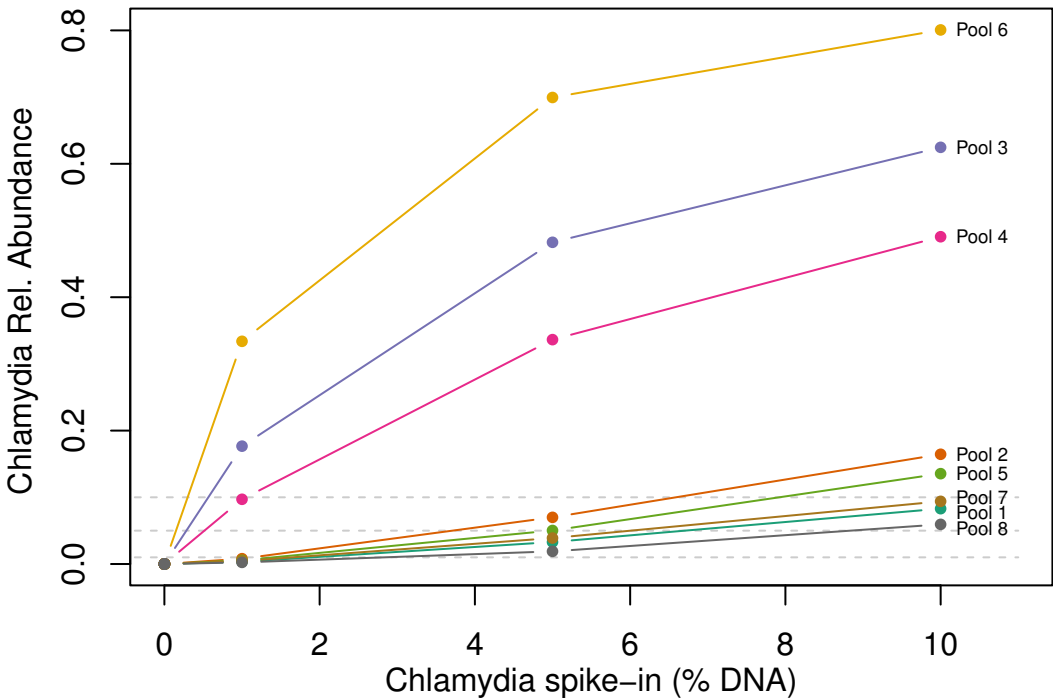

Supplement: FIG S3 [file mSphere.00448-20-sf003.pdf]
